# Correlation of Thyroid Imaging Reporting and Data System [TI-RADS] and fine needle aspiration: experience in 1,000 nodules

**DOI:** 10.1590/S1679-45082016AO3640

**Published:** 2016

**Authors:** Antonio Rahal, Priscila Mina Falsarella, Rafael Dahmer Rocha, João Paulo Bacellar Costa Lima, Matheus Jorge Iani, Fábio Augusto Cardillo Vieira, Marcos Roberto Gomes de Queiroz, Jairo Tabacow Hidal, Miguel José Francisco, Rodrigo Gobbo Garcia, Marcelo Buarque de Gusmão Funari

**Affiliations:** 1 Hospital Israelita Albert Einstein, São Paulo, SP, Brazil.

**Keywords:** Thyroid nodule/classification, Biopy, fine-needle, Thyroid gland/ultrasonography, Thyroid gland/citology

## Abstract

**Objective:**

To correlate the Thyroid Imaging Reporting and Data System (TI-RADS) and the Bethesda system in reporting cytopathology in 1,000 thyroid nodules.

**Methods:**

A retrospective study conducted from November 2011 to February 2014 that evaluated 1,000 thyroid nodules of 906 patients who underwent ultrasound exam and fine needle aspiration.

**Results:**

A significant association was found between the TI-RADS outcome and Bethesda classification (p<0.001). Most individuals with TI-RADS 2 or 3 had Bethesda 2 result (95.5% and 92.5%, respectively). Among those classified as TI-RADS 4C and 5, most presented Bethesda 6 (68.2% and 91.3%, respectively; p<0.001). The proportion of malignancies among TI-RADS 2 was 0.8%, and TI-RADS 3 was 1.7%. Among those classified as TI-RADS 4A, proportion of malignancies was 16.0%, 43.2% in 4B, 72.7% in 4C and 91.3% among TI-RADS 5 (p<0.001), showing clear association between TI-RADS and biopsy results.

**Conclusion:**

The TI-RADS is appropriate to assess thyroid nodules and avoid unnecessary fine needle aspiration, as well as to assist in making decision about when this procedure should be performed.

## RESUMO

**Objetivo:**

Apresentar a correlação entre o *Thyroid Imaging Reporting and Data System* (TI-RADS) e o sistema Bethesda, para relatar citopatologia em 1.000 nódulos tireoidianos.

**Métodos:**

Estudo retrospectivo realizado no período de novembro de 2011 a fevereiro de 2014, que avaliou 1.000 nódulos tireoidianos de 906 pacientes submetidos a exame de ultrassonografia e à punção aspirativa por agulha fina.

**Resultados:**

Observou-se associação significativa entre o TI-RADS e o resultado da classificação de Bethesda (p<0,001). A maioria dos indivíduos com TI-RADS 2 ou 3 teve resultado citológico Bethesda 2 (95,5% e 92,5%, respectivamente). Entre aqueles classificados TI-RADS 4C e 5, a maioria teve resultado Bethesda 6 (68,2% e 91,3%, respectivamente; p<0,001). A proporção de malignidades em TI-RADS 2 foi 0,8% e em TI-RADS 3 foi 1,7%. Entre TI-RADS 4A, foi de 16,0%, 43,2% em 4B, 72,7% em 4C e em 5 foi de 91,3% (p<0,001), mostrando clara associação entre o TI-RADS e os resultados da biópsia.

**Conclusão:**

O TI-RADS é apropriado para avaliar nódulos da tireoide e evitar punção aspirativa por agulha fina desnecessária, além de auxiliar na decisão sobre quando este procedimento deve ser realizado.

## INTRODUCTION

Thyroid nodules are very prevalent – they are found in approximately 8% of adults by palpation, 41% by means of ultrasound (US), and in 50% in autopsy pathological examination.^(1)^ Thyroid malignancy is relatively rare, and it is diagnosed in approximately 10% of all thyroid nodules.^(2-4)^ The appropriate indication of which nodules should undergo fine needle aspiration (FNA) and which can be monitored is still under debate. In the past two decades several controversies regarding the malignant characteristics aroused, but there is no definitive classification yet.^(1,5-7)^

In the last 5 years, some publications have tried to establish a reliable guideline for thyroid nodule sonographic evaluation.^(3-5,8,9)^ Based on the already established *Breast Imaging Reporting and Data System* ^®^ (BI-RADS^®^) for breast nodules,^(10)^ some reports suggested a categorization system of US features in thyroid nodules - the Thyroid Imaging Reporting and Data System (TI-RADS). The purpose of TI-RADS is to group the nodules into different categories with a similar percentage of malignancy as in BI-RADS^®^. It is based on thyroid nodules classification, exclusively regarding B-mode ultrasonographic features, to reduce inter-observer and inter-device variability.

## OBJECTIVE

To present the results of our initial experience in the correlation between TI-RADS and the Bethesda system for cytopathology reports of 1,000 thyroid nodules in patients who underwent sonographic evaluation, followed by fine needle aspiration, and classified according to TI-RADS system.

## METHODS

The institutional review board approved this retrospective study, and the requirement to obtain Informed Consent form was waived. From November 2011 to February 2014, US scan of thyroid gland and neck area and US-guided FNA of thyroid focal nodules were performed by experienced physicians, in our intervention center. The Medical Ethics Committee of the organization approved the study under protocol number CAAE: 41699015.8.0000.0071.

A total of 1,000 thyroid nodules in 906 patients were analyzed and classified according to TIRADS, without prior knowledge of the cytological results. The US equipment used were the ATL HDI 5000 (Absolute Medical Equipment, Wesley Hills, New York, United States), IU 22 Philips (Philips Healthcare, Andover, Massachusetts, United States), Aplio 500 Platinum (Toshiba American Medical Systems, Tustin, California, United States) and My Lab 75 (Esaote, Genova, Italy), and the acquired images stored in the PACS System (Carestream Health, California, United States).

**Figure 1:**
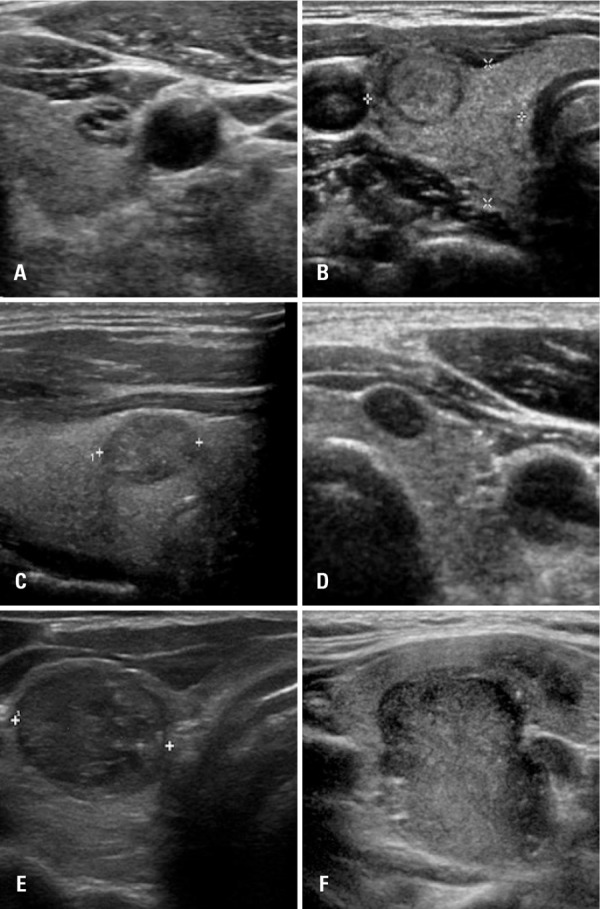
Examples of thyroid nodules submitted to cytological examination. (A) Nodule classified as TI-RADS 2; (B) TI-RADS 3; (C) TI-RADS 4A; (D) TI-RADS 4B; (E) TI-RADS 4C; (F) TI-RADS 5; cases A, B and C were considered benign; cases D, E and F were consider malignant according to Bethesda system(11)

The TI-RADS classification ranged from 1 (negative findings) to 6 (known proved malignancy) and category 4 was further divided into subcategories 4A (low suspicion), 4B (intermediate suspicion) and 4C (moderate suspicion). The higher the grade of the nodule, the greater the risk of malignancy is (Table 1 and Figure 1).

**Table 1:** Thyroid Imaging Reporting and Data System (TI-RADS) classification

TI-RADS	Definition	Ultrasound features
1	Negative	Normal thyroid
2	Benign	Benign features
3	Probably benign	Without suspicious features
4A	Low suspicion	One suspicion feature
4B	Intermediate suspicion	Two suspicion features
4C	Moderate suspicion	Three or four suspicion features
5	High suspicion	Five suspicion features
6	Known proved malignancy	Confirmed malignancy

The US features associated to higher malignancy risks were irregular margins, hipoechogenicity, marked hypoechogenicity (defined as solid nodules, without enhancement or spots, with areas of greater hypoechogenicity within the nodule, or in relation to other hypoechoic areas of the thyroid gland; in this situation, we considered both points), morphology longer than wide, and microcalcifications.

FNA was performed by freehand technique under US guidance, using a 23-gauge needle attached to a 20cc syringe. Upon aspiration, a negative pressure was maintained until blood appeared in the hub of the syringe. The anesthesia used was the combination of local anesthetic (lidocaine) and ice. Crossing thyroid vessels was avoided to prevent local bleeding; in mixed nodules, solid areas were chosen.

In all FNA procedures, prior to patient discharge, a cytologist assessed the sample to avoid unnecessary punctures and insufficient specimens. After this initial evaluation, experienced pathologists evaluated all samples according to Bethesda system (Table 2).

**Table 2:** The Bethesda System for Reporting Thyroid Cytopathology

Category	Meaning
I	Non-diagnostic or inadequate
II	Benign
III	Atypia/follicular lesion of undetermined significance
IV	Follicular neoplasm or suspicious for follicular neoplasm
V	Suspicious for malignancy
VI	Malignant

Source: Cibas et al.^(11)^

The relation between TI-RADS and Bethesda was evaluated through double entry tables, χ^2^ and Pearson’s correlation tests. Considering the biopsy results as malignant or benign, to analyze the association we used binary logistic regression models and assessed the odds ratios of malignancy for each TI-RADS category. The odds ratios were expressed using 95% confidence interval (95%CI). The tests were performed using the Statistical Package for Social Science (SPSS) for Windows, version 17.0, and considering the significance level (α) of 5%.

## RESULTS

One thousand examinations of 906 patients were carried out. Of the total of 1,000 examinations, 24 were Bethesda I and were excluded; hence, we had 976 complete examinations. The nodules classified as Bethesda cytology IV, V and VI were considered suspicious for malignancy.

Taking into account all nodules in the analysis, a significant association was observed between the TI-RADS and the Bethesda (p<0.001) classification, and those with TI-RADS rating 2 or 3 were mostly Bethesda 2 (95.5% and 92.5%, respectively). Among those classified as TI-RADS 4C and 5 (68.2% and 91.3%, respectively), the majority was Bethesda 6 (Table 3).

**Table 3:** Thyroid Imaging Reporting and Data System (TI-RADS) and Bethesda correlation

TI-RADS classification	Bethesda results					

	2	3	4	5	6	Total
All nodules	n (%)	n (%)	n (%)	n (%)	n (%)	n (%)
2	120 (96)	4 (3.2)	0 (0)	0 (0)	1 (0.8)	125
3	432 (93.3)	23 (5)	0 (0)	0 (0)	8 (1.7)	463
4A	192 (73.3)	28 (10.7)	6 (2.3)	1 (0.4)	35 (13.4)	262
4B	35 (43.2)	11 (13.6)	3 (3.7)	1 (1.2)	31 (38.3)	81
4C	3 (13.6)	3 (13.6)	1 (4.5)	0 (0)	15 (68.2)	22
5	1 (4.3)	1 (4.3)	0 (0)	0 (0)	21 (91.3)	23

Total	783 (80.2)	70 (7.2)	10 (1)	2 (0.2)	111(11.4)	976

**Figure 2:**
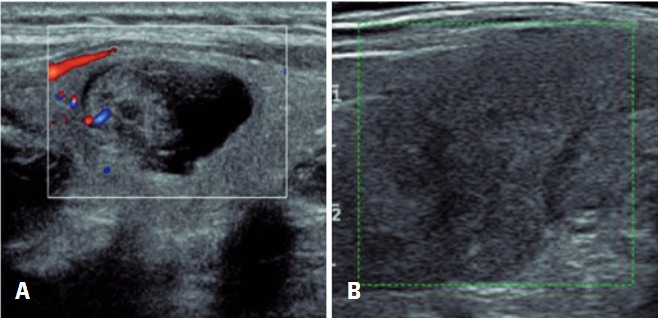
Thyroid ultrasound with unexpected results. (A) Nodule classified as TI-RADS 2 that showed malignancy in cytological examination. (B) Nodule classified as TI-RADS 5 with granulomatous thyroiditis in cytological examination (Bethesda II)

There was an unexpected malignancy case in a nodule that had been classified as TI-RADS 2. A retrospective revision of the images showed that the nodule, in fact, should have been classified as 3 (Figure 2A). Probably the nodule was misclassified by wrongly considering the solid part as spongiform and, in fact, it did not contain colloid foci. There was also one nodule classified as TI-RADS 5 that was benign. It was a case of thyroiditis, confirmed in follow-up US exams (Figure 2B). All the remaining percentages of malignancy in cytology were similar to BI-RADS^®^ method, widely accepted and established.

In the analysis considering all nodules, the proportion of malignant nodules classified as TI-RADS 2 was 0.8%, and among those TI-RADS 3, it was 1.7%. We classified as 4A 16%, 43.2% as 4B, 72.7% as 4C classification, and 91.3% as TI-RADS 5. The results of the logistic regression model was p<0.001, showing a clear association between TI-RADS and biopsy results. The group rated TI-RADS 3 was considered as the reference for the model to be more numerous. The risk of malignancy for patients classified 4A was estimated at 10.86-fold the risk for those rated as 3 (95%CI: 5.0-23.5). For the individuals with 4B classification, the risk of malignancy was estimated to be 43.27-fold that for patients rated 3 (95% CI: 18.95-98.92). The other estimated odds ratios are presented in table 4.

**Table 4:** Thyroid Imaging Reporting and Data System (TI-RADS) and correlation with risk of malignancy

TI-RADS All nodules	Malignancy			OR 95%CI	p value

	Benign	Malignant	Total		
		
	n (%)	n (%)	n (%)		
2	124 (99.2)	1 (0.8)	125	0.46 (0.06-3,7)	0.464
3	455 (98.3)	8 (1.7)	463	Reference	Reference
4A	220 (84)	42 (16)	262	10.86 (5-23.52)	0.002
4B	46 (56.8)	35 (43.2)	81	43.27 (18.9-98.82)	<0.001
4C	6 (27.3)	16 (72.7)	22	151.67 (47-488.68)	<0.001
5	2 (8.7)	21 (91.3)	23	597.19 (119.3-2987.7)	<0.001

Total	853 (87.4)	123 (12.6)	976		

95%CI: 95% confidence interval; OR: *odds ratio*.

## DISCUSSION

Thyroid US should be performed in the initial assessment of the gland.^(12,13)^ The FNA is an inexpensive and useful tool for detecting thyroid cancer, but it is an invasive procedure. In the management of thyroid nodule patients, recommending who should be submitted to FNA is still controversial. In the last decade, the improvement of Doppler US evaluation drew interest in rating thyroid nodules, based on spectrum and speed mapping parameters, which initially proved promising. In this context, the classification proposed by Chammas et al*.* ^(5)^ emerged as one of the main methods used. However, the methodology has some limitations, including much variability inter-examiners and inter-devices, which is greater in evaluation by Doppler as compared to B-mode parameters. Similarly, the retrospective analysis of images is greatly compromised. Several classifications based on US features have been proposed in the last decade, in an attempt to facilitate this selection. However a consensus has not been established, given the difficulty of reproducibility of different classifications proposed, or even due to the low correlation between the US reports and cytology results.^(3-5,8,9,12)^

Currently there is a tendency to standardize the imaging evaluation of different organs^(10,14,15)^ with reliable and easily reproducible classifications. The main example is the already established BI-RADS^®^ classification for breast nodules.

The TI-RADS classification aims to correlate US features to cytological classification, increasingly graduating the risk of a nodule being malignant, according to the number of features present in the US. Among diverse classifications, Horvath et al.,^(3)^ by means of a prospective analysis, proposed ten US patterns to be analyzed during the examination and nodule classification from TI-RADS 2 to 6 (category 4 divided into 4A and 4B) and estimated a malignancy risk of 14.1% in TI-RADS 3, 45% in TI-RADS 4, and 89.6% in TI-RADS 6. Kwak et al.^(4)^ proposed a TI-RADS classification through retrospective analysis of patients submitted to thyroid US and FNA, considering the risk of malignancy and subdivisions similar to the BI-RADS^®^ classification (that is, with three subdivisions for category 4), using five US criteria that can be added during thyroid evaluation. This article also described that a malignancy risk lower than 3% is expected for TI-RADS 3, a risk of 3.6 to 91.9% for TI-RADS 4, and of 88.7 to 97.9% for TI-RADS 5.

The present study has differences in relation to that proposed by Horvath et al.,^(3)^ such as being retrospective and with one more subdivision in the category 4, by adding 4C. Besides our purpose was to facilitate the classification process, reducing from ten to only four features in B-mode US considered in our classification. It also differed from the study by Kwak et al.^(4)^ in this issue, since these authors used five features in the classification, one more than ours. This difference relied on the nodule composition, that we judged as liable to mistakes in some cases, since many mixed nodules could generate uncertainty about their precise composition in ultrasonographic evaluation. Instead, we considered two points of marked hypoechogenicity, because the nodules with such characteristics have an increased risk of malignancy as compared to those slightly hypoechoic.^(16,17)^

Finally, our classification, unlike others proposed, does not use the sum of points for Doppler features. Although Doppler mapping help in thyroid US evaluation,^(5,9,18)^ its large-scale reproducibility is compromised due to the inter-examiner and inter-device variability.^(19)^

This study has some limitations, such as being retrospective, sonographic assessment performed by different operators and diverse US machines, use of cytology data instead of pathological data despite high sensitivity and specificity of cytology. Another limitation was the lack of uniformity of criteria for indicating puncture of nodules.

## CONCLUSION

TI-RADS can be considered an appropriate classification in the assessment of thyroid nodules, in order to avoid unnecessary fine needle aspirations and to assist in making decision about when it should be performed. This classification improves communication and reduces confusion among physicians and patients. Our experience demonstrated that the TI-RADS classification is highly reproducible, since it is based on B-mode characteristics of the nodules, especially when performed by experienced radiologists, acquainted with its use.
